# Functional characterization of a type 2 metallothionein gene, *SsMT2*, from alkaline-tolerant *Suaeda salsa*

**DOI:** 10.1038/s41598-017-18263-4

**Published:** 2017-12-20

**Authors:** Shumei Jin, Chang Xu, Guoliang Li, Dan Sun, Ying Li, Xinwang Wang, Shenkui Liu

**Affiliations:** 1 Key Laboratory of Saline-alkali Vegetation Ecology Restoration in Oil Field (SAVER), Ministry of Education, Alkali Soil Natural Environmental Science Center (ASNESC), Northeast Forestry University, Harbin, 150040 China; 2 USDA-ARS, Southern Plains Agricultural Research Center, College Station, TX 77845 United States; 3 Institute of Maize, Heilongjiang Academy of Agricultural Sciences, Harbin, 150086 China; 4 Institute of Vegetables and Flowers, Chinese Academy of Agricultural Sciences, Beijing, 100081 China

## Abstract

A type 2 metallothionein gene, *SsMT2*, was cloned from *Suaeda salsa*, a salt- and alkali-tolerant plant, which is dominant species on the saline/alkali soil of northeast China. The *SsMT2 g*ene was expressed in all organs except the flower and its expression was induced by various stresses such as CdCl_2_, NaCl, NaHCO_3_, and H_2_O_2_ treatments. SsMT2-transgenic yeast (*Saccharomyces cerevisiae*) and plants (*Arabidopsis thaliana*) showed significantly increased resistance to metal, salt and oxidant stresses. These transgenics accumulated more Cd^2+^, but less Na^+^ than their wild type counterparts. *SsMT2* transgenic *Arabidopsis* maintained lower level of H_2_O_2_ than wild type plants did in response to the stress treatments. These results demonstrated that the *SsMT2* gene plays an important role in reactive oxygen species scavenging and confers enhanced metal and oxidant tolerance to plants.

## Introduction

Saline-alkaline soils are widely distributed on earth, and the total global area of salt-affected soils, including saline-alkaline soils, is 8.31 × 10^9^ ha^1^. The saline-alkaline soils in Northeast China contain a high concentration of NaHCO_3_^2^. Very few plants survive in this area, and those that do have high tolerance to saline/alkaline stress. The genus *Suaeda* consists of 110 species of which most are highly salt tolerant^3,4^. In saline/alkaline communities of northeast China, *S*. *salsa* is typically the predominant vegetation. *S*. *salsa* accumulates salts within cells, therefore, significantly decreases the salt concentrations in the soil^5^. At a density of 15 plants/m^2^, *S*. *salsa* plants can remove 303–386 g/m^2^ of Na^+^ from saline soil during its growing season, which suggests that *S*. *salsa* could be used to improve the saline soil quality^6^. Several *S*. *salsa* communities have been developed as tourism resources in saline-alkali soil^7^. *S*. *salsa* also can regulate transportation or transformation of nutrients and heavy metals^8^. Because *S*. *salsa* can survive in soil with high NaHCO_3_ content, it may have a special mechanism to accommodate the formidable salt/alkali in the environment.

An extensive number of studies has been completed in plants addressing tolerance to salinity and/or alkalinity, leading to identification of a class of plant Metallothioneins (MTs) proteins, that are associated with plant resistance extreme environmental stress^9^. MTs are a family of low molecular weight (7–10 kDa), Cys-rich proteins that bind to metals in a range of organisms, such as *Oryza sativa* ^10^, *Arabidopsis* ^11^, *Elsholtzia haichowens is* ^12^, and *Gossypium hirsutum* ^13^. MTs are divided into three classes based on the arrangement of Cys residues^14^. Plant MTs belong to class II and can be further subdivided into the following four types: MT1, MT2, MT3, and MT4, based on the Cys distribution pattern^15^.

MT function in plants can be triggered when plants suffer metal and/or salt stress. Several MT genes have been cloned. For example, *EhMT1* was cloned from *E*. *haichowensis* under high Cu^2+^concentration^16^, *Hordeum vulgare MT* from Fe-deficient roots^17^, *Triticum aestivum MT* from roots treated with Al^3+^ ^18^, tomato *MT* and cabbage *MT* from roots treated with Cd^2+^ ^19,20^, *Silene nicaeensis SnMT2* from root of plants collected from area with higher metal pollution index (MPI)^21^, *Oryza sativa rgMT* and *Chloris virgata Swartz ChlMT1* from seedlings treated with NaHCO_3_^22–24^, and celery *pAgMT2* and *pAgMT3* were induced by salt stress^25^.

The ectopic expression of *OsMT1e-P* enhanced tolerance of salt stresses in transgenic tobacco, and the resultant plants survived and set viable seeds under saline conditions^26^. A *SbMT2* gene was used to transform tobacco, and transgenic lines had better phenotypic performance under salt (NaCl) stress conditions compared to wildtype plants^27^. Overexpression of *OsIFL* in transgenic tobacco plants conferred salinity stress tolerance. Screening of a rice cDNA library revealed *OsIFL* strongly interacted with metallothionein protein^28^.

Cadmium (or Cd^2+^), among the most toxic non-essential elements with high mobility in plants, directly or indirectly inhibits primary physiological processes^29^. The photosynthetic apparatus appears to be particularly sensitive to Cd^2+^ toxicity, even at very low concentrations^30^. MTs were first isolated as Cd-binding protein from horse kidney in 1957^31^. This family of proteins detoxifies metal ions through direct binding Cd^2+^ ^9^.

The production of reactive oxygen species (ROS) occurs at all times during plant growth and development^32^, and increases when plants are exposed to biotic and abiotic stresses^33^. The cysteines in MTs directly involved in the removal of ROS and thus, protect against cellular injury, and indirectly reduce the production of cellular ROS^34^. MTs may act as an antioxidant by mitigating ROS-induced cellular injury independent of a function in metal sequestration^35^.

Each kind of MT may have a unique function and plays an important role against abiotic stress. Because *S*. *salsa* grows in saline or alkaline soil habitat and persists^3,4^, the biological function of MTs in anti-alkali plants has not been elucidated. Therefore, we cloned an open reading frame of a type 2 MT, designated as *SsMT2*, from *S*. *salsa* and investigated its function under the stress induced by Cd^2+^, Na^+^ and H_2_O_2_ in transgenic yeast (*Saccharomyces cerevisiae*) and *Arabidopsis thaliana*. The results enhance our insights into the *SsMT2* gene function when halophyte plants are grown under environmental stresses.

## Results

### Cloning of an open reading frame of *SsMT2* in *S*. *salsa*

The open reading frame (ORF) of *SsMT2* was obtained from the cDNA in the *S*. *salsa*. The full-length fragment contains of 234 bp and encodes a 77-amino acid polypeptide GenBank accession number MF447531). The amino acid sequence of this transcript had the highest similarity (91%) with that of the *SbMT* protein (GenBank accession number: JF780913) from *Salicornia brachiata*, followed by *AcMT* from *Amaranthus cruentus* (AF268027) (79%), *SnMT* from *Silene niceensis* (ADP92404) (75%), and *SmMT* from *Salvia miltiorrhiza* (ABR92329) (60%) (Fig. S1).

### *SsMT2* gene expression in *S*. *salsa*

**Figure 1:**
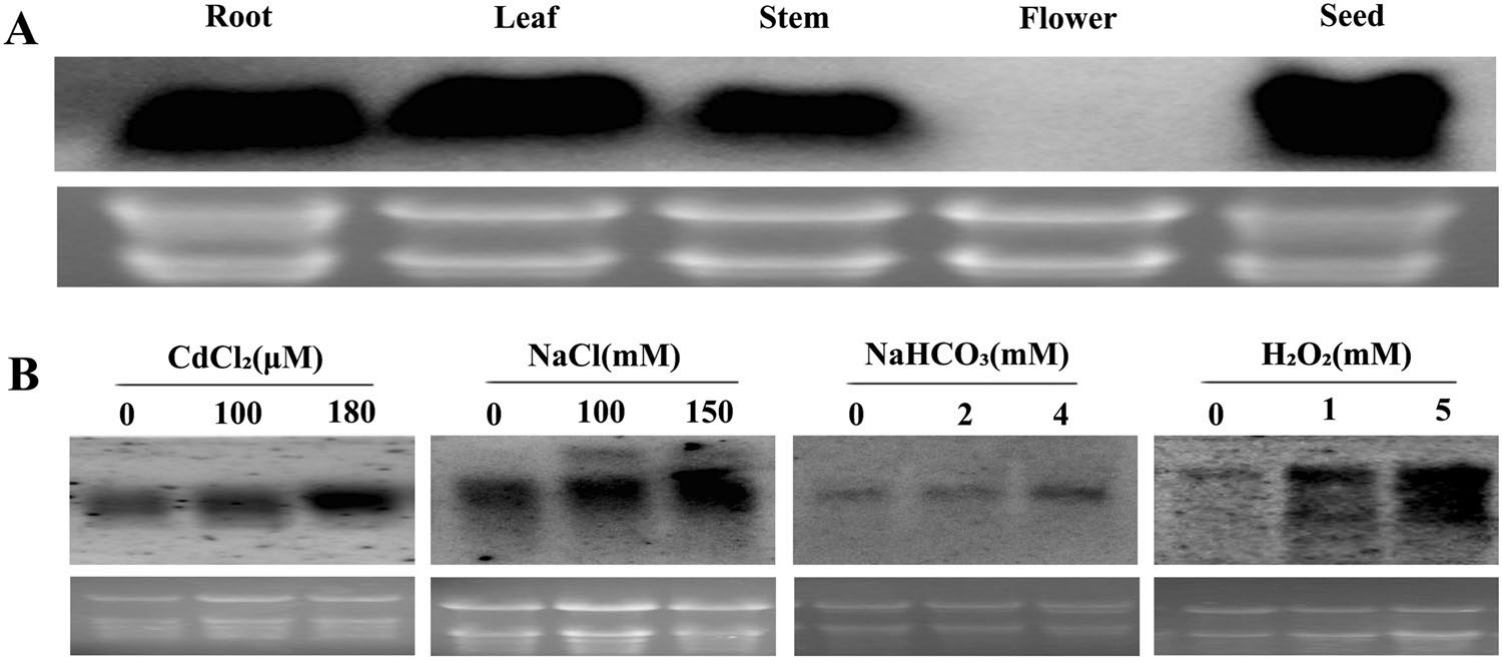
Organ distribution of *SsMT2* expressionin *S*. *salsa* and detection of *SsMT2*transcripts in stress-treated *S*. *salsa*. (**A**) Northern blot analysis showed the differential expression of *SsMT2* in different organs of *S*. *salsa*. (**B**) Gene expression in *S*. *salsa* after different stresses treatments for 48 h showed in Northern blot. No treatment (CK = 0) is a control. Cropped images were displayed and original blots are shown in the Supplementary 3.

Northern blot detected strong signals in roots, leaves, stems and seed, but no signal in flowers, indicating that the *SsMT2* gene expressed in all organs except flowers (Fig. 1A). The expression of the *SsMT2* gene was significantly induced under CdCl_2_ and H_2_O_2_ stresses in *S*. *salsa*. NaCl stress caused a moderate increase of the transcript and NaHCO_3_ stress caused slight increase of the transcript (Fig. 1B). The results indicated that different stresses affect the *SsMT2* expression differentially in *S*. *salsa*.

### *SsMT2*-transgenic yeast responses to Cd^2+^, Na^+^ and H_2_O_2_ stresses

Northern blot showed that one distinct band was detected in the transgenic yeast and no signal in the control and indicated that the *SsMT2* gene was expressed in the transgenic yeast (Fig. S2A). The quantification of SsMT2 protein in yeast was analyzed using Western blot (Fig. S2B). Stronger signals were detected in *SsMT2* transformed yeast, compared to weak signal in WT yeast (non-*SsMT2* transformed). This result indicated that some other MT proteins present in the yeast, and *SsMT2* transformed yeast has more MT protein than WT yeast.

**Figure 2:**
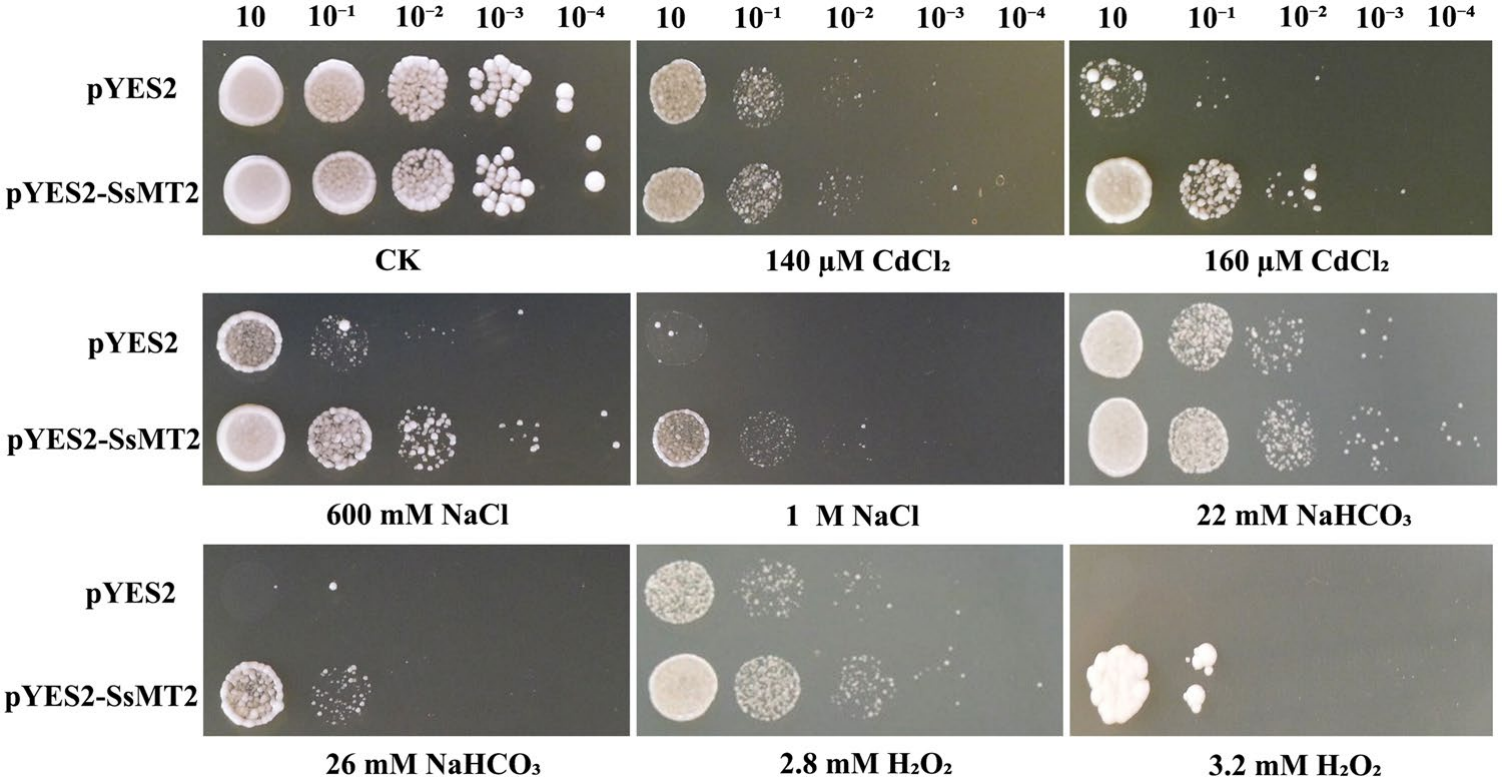
Growth of *SsMT2*-overexpressed yeast cells under stress condition. Ten-fold dilutions of yeast cells containing pYES2 (upper line) and pYES2-SsMT2 vector (lower line) were spotted onto solid YPG media supplemented with the indicated stresses and grew at 30 °C for 3–7 d. No treatment is a control (CK).

The cell growth of transgenic and non-transgenic yeasts was compared at five serial dilutions for each treatment (corresponding to the five columns in each panel in Fig. 2). Without stress (control), the growth of both transgenic and non-transgenic yeasts showed no significant difference (upper left panel in Fig. 2). However, growth was affected when the stresses were applied. The transgenic yeasts grew better than the non-transgenic yeasts in the presence of 140 µM CdCl_2_, 600 mM NaCl, 22 mM NaHCO_3_ or 2.8 mM H_2_O_2_ (Fig. 2). When the concentration was increased to 160 µM CdCl_2_, 1 M NaCl, 26 mM NaHCO_3_, or 3.2 mM H_2_O_2_, the transgenic yeasts grew, but non-transgenic yeasts did not grow (Fig. 2).

### *SsMT2*-transgenic *Arabidopsis* responses to Cd^2+^, Na^+^ and H_2_O_2_ stresses

The copy numbers of the *SsMT2* gene in the transgenic lines were indicated by one or more distinct bands in the transgenic *Arabidopsis*. There were four plants (#1, #3, #5 and #6 in Fig. S2C) that had one copy, one plant (#2) that had three copies (Fig. S2C), and one plant (#4) that had nine copies (Fig. S2C). No positive signal was detected in WT *Arabidopsis* plants (Fig. S2C). The expression of *SsMT2* gene in transgenic *Arabidopsis* was detected by Northern blot. Of these transgenic *Arabidopsis* plants, three (#1, #5 and #6 in Fig. S2D) were positive, and indicated the *SsMT2* gene was highly expressed in these transgenic plants.

**Figure 3:**
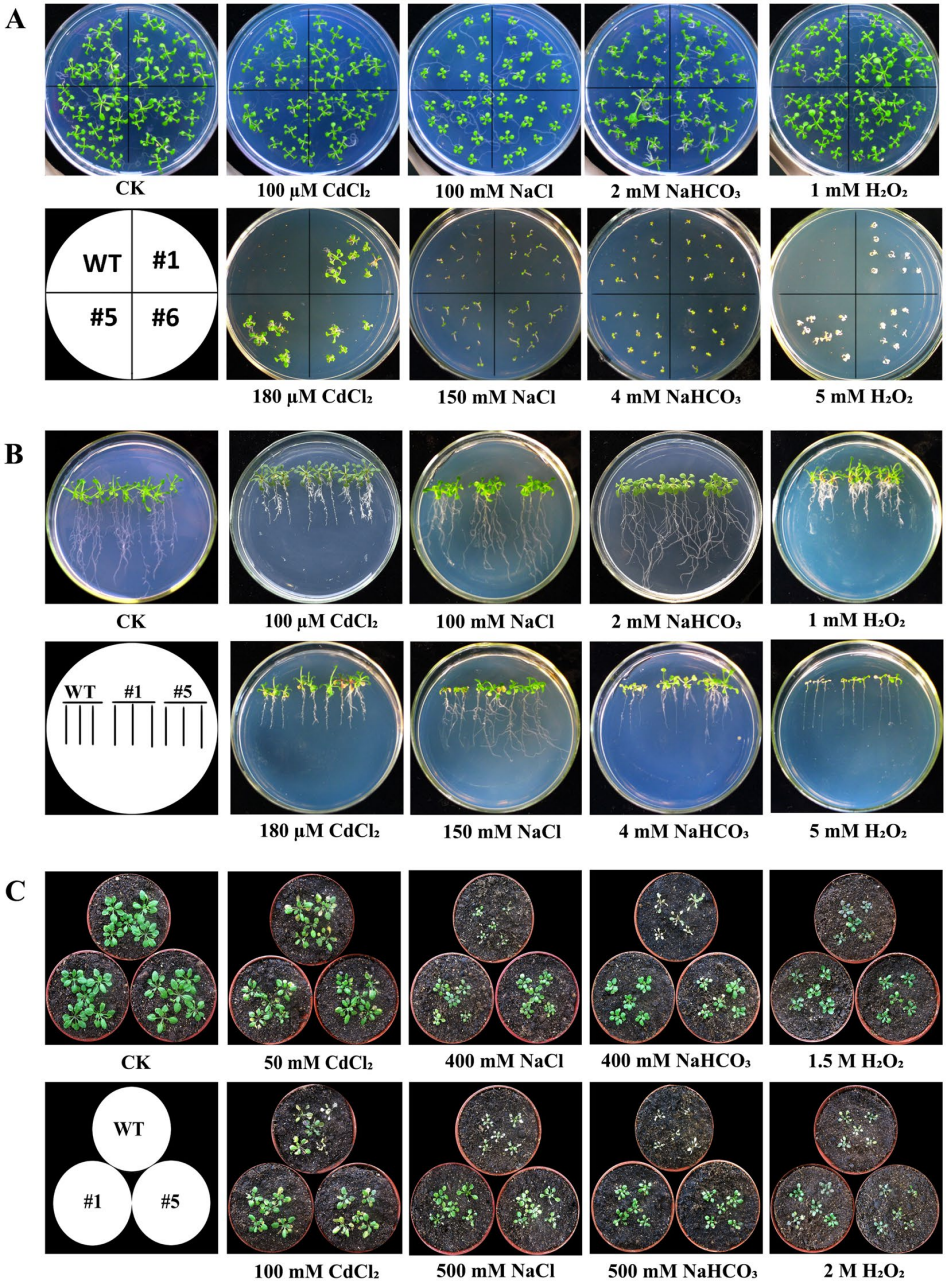
Seed germination and plants growth of transgenic plants under different stresses. (**A**) Seed germination on medium supplemented with 0 (CK), 100 µM CdCl_2_, 180 µM CdCl_2_, 100 mM NaCl, 150 mM NaCl, 2 mM NaHCO_3_, 4 mM NaHCO_3_, 1 mM H_2_O_2_ or 5 mM H_2_O_2_ in the *Arabidopsis* wild type (WT) and transgenic plants (#1, #5, #6). (**B**) Relative stress tolerance of WT and *SsMT2*-overexpressed third generation transgenic *Arabidopsis* plants (#1 and #5) at the seedling stage. 14-day-old seedlings were grown on medium supplemented each of 0 (CK), 100 µM CdCl_2_, 180 µM CdCl_2_, 100 mM NaCl, 150 mM NaCl, 2 mM NaHCO_3_, 4 mM NaHCO_3_, 1 mM H_2_O_2_ or 5 mM H_2_O_2_. C. Relative stress tolerance of wild type and *SsMT2*-overexpressed third generation transgenic *Arabidopsis* plants (#1 and #5) at the adult stage. 28-day-old plants were grown on soil supplemented each of 0 (CK), 50 mM CdCl_2_, 100 mM CdCl_2_, 400 mM NaCl, 500 mM NaCl, 400 mM NaHCO_3_, 500 mM NaHCO_3_, 1.5 M H_2_O_2_ or 2 M H_2_O_2_.

The effects of CdCl_2_, NaCl, NaHCO_3_ and H_2_O_2_ on seed germination were examined in the above three selected transgenic *Arabidopsis* and wild type plants (Fig. 3A). Seeds of wild type and transgenic plants were germinated on medium, each containing 100 µM CdCl_2,_ 100 mM NaCl, 2 mM NaHCO_3_ or 1 mM H_2_O_2_, with 3 days later for wild type than transgenic lines. In the presence of 150 mM NaCl or 4 mM NaHCO_3_, only 40% or 20% wild type lines seed germination respectively, while 100% transgenic lines seed germination. In the presence of 180 µM CdCl_2_ or 5 mM H_2_O_2_, no wild type seeds were germinated. Although transgenic plant seeds were also heavily affected, 48% or 72% seeds were germinated respectively. The transgenic lines extended germination until the cotyledon turned white under 5 mM H_2_O_2_. On the control (no stress) media, seed germination showed no significant difference between wild type and three selected transgenic lines (Fig. 3A).

The effects of CdCl_2_, NaCl, NaHCO_3_ and H_2_O_2_ on seedling growth were examined at the early stage of growth of transgenic plants #1 and #5 (#5 and #6 had very similar phenotype, so only #5 plant was selected for analysis) (Fig. 3B). No significant phenotypic difference was observed between the transgenic lines and WT plants on the control medium. However, the growth of transgenic and WT lines was inhibited when the medium contained 100 µM CdCl_2_, 100 mM NaCl, 2 mM NaHCO_3_, or 1 mM H_2_O_2_. However, the transgenic plants grew better than their WT counterparts. The growth of young leaves of the *SsMT2*-transgenic lines were less affected under the 180 µM CdCl_2_, 150 mM NaCl, or 4 mM NaHCO_3_ stress compared to the wild type plants. There were no significant differences in the dry weights of the *SsMT2* transgenic lines and WT plants without stresses and 5 mM H_2_O_2_. However, green leaves in transgenic plant and white leaves in WT plants were observed when grown on the medium with 5 mM H_2_O_2_. Dry weight (Table 1) of the *SsMT2* transgenic lines was higher than WT plants under other stress conditions. Additionally, there was no significant difference with root length among plants between transgenic and wild type plants under stress (data not shown). These results showed that the *SsMT2* gene expression in *Arabidopsis* transgenic plants increased metal, salt or oxidant tolerance during early stage of seedling growth.

**Table 1:** Dry weigh (mg/10 plants) of *Arabidopsis* under different stress treatments. Results are presented as means ± SE (n = 3). Low case letters a and b indicate significant differences among mean values within each plant at p ≤ 0.05. CK, control; WT, wild type; #1 and #5 are *SsMT2*- transgenic plants.

Plant	CK	CdCl_2_		NaCl		NaHCO_3_		H_2_O_2_	
		100 μM	180 μM	100 mM	150 mM	2 mM	4 mM	1 mM	5 mM
WT	14.1 ± 0.8	7.2 ± 0.5^a^	2.1 ± 0.1^a^	8.7 ± 0.4^a^	1.9 ± 0.1^a^	8.8 ± 0.5^a^	1.9 ± 0.1^a^	7.6 ± 0.6^a^	1.6 ± 0.1
#1	14.8 ± 1.0	10.6 ± 0.9^b^	4.8 ± 0.3^b^	10.3 ± 1.0^b^	5.2 ± 0.4^b^	11.2 ± 0.8^b^	3.9 ± 0.2^b^	9.8 ± 0.9^b^	1.8 ± 0.3
#5	14.4 ± 0.7	11.1 ± 1.0^b^	5.9 ± 0.6^b^	11.4 ± 0.9^b^	5.4 ± 0.4^b^	12.4 ± 0.9^b^	3.8 ± 0.3^b^	10.2 ± 1.2^b^	1.9 ± 0.3

The effects of CdCl_2_, NaCl, NaHCO_3_ and H_2_O_2_ on #1 and #5 transgenic plants were examined during adult stage of plant growth (Fig. 3C). No phenotypic differences were observed between the transgenic and WT plants under normal conditions. After exposing both sets of plants to 100 mM CdCl_2_, 400 mM NaCl, 500 mM NaCl, 400 mM NaHCO_3_ or 500 mM NaHCO_3_, 1.5 M H_2_O_2_, or 2 M H_2_O_2_ stress, *SsMT2*-transgenic plants had a significantly higher survival rate than WT plants (Table 2).

**Table 2:** Survived rate under different stress treatments. Results are presented as means ± SE (n = 3). Low case letters a and b indicate significant differences among mean values within each plant at p ≤ 0.05. CK, control; WT, wild type; #1 and #5 are *SsMT2*- transgenic plants.

Plant	CK	CdCl_2_		NaCl		NaHCO_3_		H_2_O_2_	
		50 mM	100 mM	400 mM	500 mM	400 mM	500 mM	1.5 M	2 M
WT	100%	100%	66.67%^a^	73.33%^a^	33.33%^a^	26.67%^a^	6.67%^a^	93.33%^a^	40%^a^
#1	100%	100%	93.33%^c^	93.33%^b^	66.67%^b^	100%^c^	73.33%^b^	100%^b^	86.67%^b^
#5	100%	100%	86.67%^b^	100%^c^	80%^c^	93.33%^b^	80%^c^	100%^b^	73.33%^c^

### Metal ion uptake in *SsMT2*-transgenic yeast

*SsMT2*-transgenic yeast accumulated higher amounts of Cd^2+^ (Table 3) and lower amounts Na^+^ (Table 4) than non-transgenic yeast (control) when exposed to 140 µM CdCl_2_, 160 µM CdCl_2_, 600 mM NaCl, 1 M NaCl, 22 mM NaHCO_3_, or 26 mM NaHCO_3_stresses. No significant differences in the amount of Cd^2+^ and Na^+^ accumulation were observed between transgenic and non-transgenic yeast on the YPG (1% yeast extract + 2% peptone + 2% galactose) medium without any stresses.

**Table 3:** Cd^2+^ accumulation (μg/g dry weight) in yeast and *SsMT*-transgenic yeast under CdCl_2_ stresses treat. Results are presented as means ± SE (n = 3). Low case letters a and b indicate significant differences among mean values within each plant at p ≤ 0.05. pYES2, yeast cell without *SsMT2*; pYES2-SsMT2, yeast cell containing *SsMT2*.

Yeast	CK	140 μM	160 μM
pYES2	31.75 ± 2.31	79.76 ± 4.32^a^	99.73 ± 8.91^a^
pYES2-SsMT2	29.12 ± 2.17	119.42 ± 20.2^b^	149.71 ± 13.12^b^

**Table 4:** Na^+^ accumulation (mg/g dry weight) in yeast and *SsMT*-transgenic yeast under NaCl or NaHCO_3_ treatment. Results are presented as means ± SE (n = 3). Low case letters a and b indicate significant differences among mean values within each plant at p ≤ 0.05. pYES2, yeast cell without *SsMT2*; pYES2-SsMT2, yeast cell containing *SsMT2*. pYES2, yeast cell without *SsMT2*; pYES2-SsMT2, yeast cell containing *SsMT2*.

Yeast	CK	NaCl		NaHCO_3_	
		600 mM	1 M	22 mM	26 mM
pYES2	69.80 ± 7.12	249.43 ± 21.21^b^	352.18 ± 21.15^b^	83.42 ± 10.1^b^	96.61 ± 8.12^b^
pYES2-SsMT2	73.51 ± 7.36	199.48 ± 17.44^a^	228.12 ± 20.17^a^	79.21 ± 7.20^a^	91.35 ± 8.13^a^

### Metal ion uptake in *SsMT2*-transgenic *Arabidopsis* plants

Cd^2+^ and Na^+^ concentrations in transgenic and WT plants were measured to determine whether or not overexpression of the *SsMT2* gene affected the Cd^2+^ and Na^+^ accumulation in transgenic *Arabidopsis* plants. On Murashige and Skoog basal (MS) medium, the concentrations of Cd^2+^ (Table 5) and Na^+^ (Table 6) in the shoots and roots did not differ significantly between transgenic and WT seedlings. The concentration of Cd^2+^ in *SsMT2*-transgenic and WT plants increased dramatically, with a relatively higher level in roots and shoots of *SsMT2*-transgenic plants when seedlings were grown on medium containing either 100 µM CdCl_2_ or 180 µM CdCl_2_. When exposed either to 100 or 150 mM NaCl, 2 or 4 mM NaHCO_3_, the Na^+^ concentrations in the transgenic and WT plants dramatically increased, but with a relatively lower level in both roots and shoots of *SsMT2*–transgenic lines.

**Table 5:** Cd^2+^ accumulation (μg/g dry weight) in shoots and roots of wild-type and transgenic *Arabidopsis* lines in the presence of CdCl_2_. Results are presented as means ± SE (n = 3). Low case letters a and b indicate significant differences among mean values within each plant at p ≤ 0.05. CK, control; WT, wild type; #1 is *SsMT*-transgenic plants.

Plant	Shoot			Root		
	CK	100 μM	180 μM	CK	100 μM	180 μM
WT	0.25 ± 0.01	1.15 ± 0.14^a^	1.72 ± 0.12^a^	0.12 ± 0.01	2.83 ± 0.25^a^	4.90 ± 0.39^a^
#1	0.20 ± 0.01	1.51 ± 0.13^b^	2.61 ± 0.21^b^	0.11 ± 0.01	3.81 ± 0.40^b^	6.91 ± 0.46^b^

**Table 6:** Na^+^ accumulation (mg/g dry weight) in shoots and roots of wild-type and transgenic *Arabidopsis* lines in the presence of NaCl or NaHCO_3_. Results are presented as means ± SE (n = 3). Low case letters a and b indicate significant differences among mean values within each plant at p ≤ 0.05. CK, control; WT, wild type; #1 is *SsMT2*-transgenic plants.

Organ	Plant	CK	NaCl		NaHCO_3_	
			100 mM	150 mM	2 mM	4 mM
shoot	WT	0.92 ± 0.01	43.10 ± 4.1^b^	55.00 ± 3.11^b^	7.32 ± 0.52^b^	8.81 ± 0.41^b^
	#1	0.88 ± 0.02	32.41 ± 2.51^a^	48.11 ± 2.12^a^	5.20 ± 0.42^a^	7.52 ± 0.31^a^
root	WT	0.62 ± 0.02	14.11 ± 1.10^b^	16.72 ± 1.20^b^	4.21 ± 0.21^b^	6.00 ± 0.52^b^
	#1	0.58 ± 0.01	5.91 ± 0.31^a^	8.31 ± 0.91^a^	2.92 ± 0.10^a^	4.01 ± 0.26^a^

### Effects of treatments on the production of H_2_O_2_ in plant leaves

**Figure 4:**
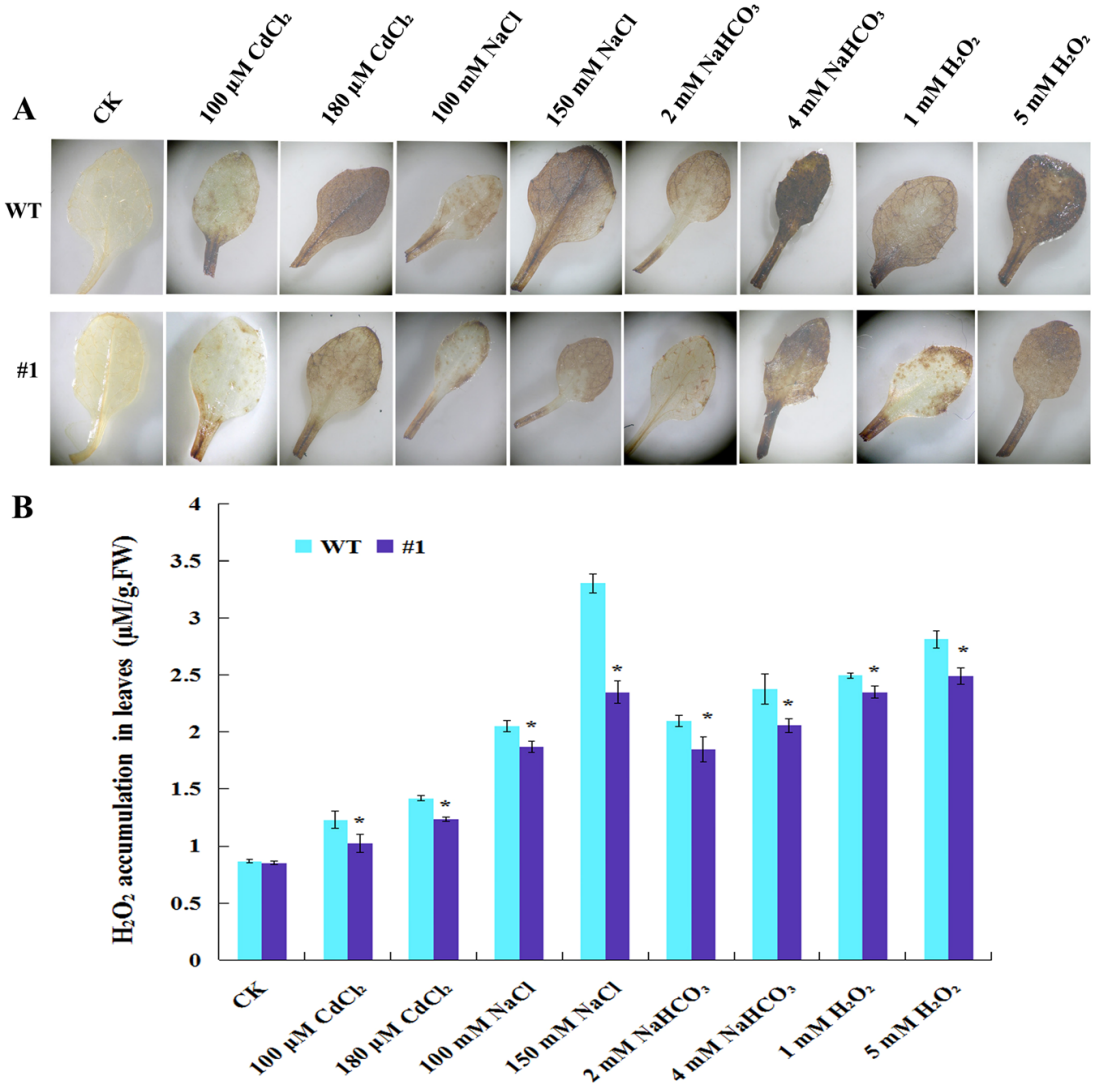
3,3′-Diaminobenzidine (DAB) staining (**A**) and H_2_O_2_ content (**B**) in leaves in wild type and transgenic *Arabidopsis* under different stresses. Seedling leaves of WT and transgenic (#1) *Arabidopsis* plants were grown on medium supplemented with no treatment (CK), 100 µM CdCl_2_, 180 µM CdCl_2_, 100 mM NaCl, 150 mM NaCl, 2 mM NaHCO_3_, 4 mM NaHCO_3_, 1 mM H_2_O_2_ or 5 mM H_2_O_2_ for 48 h. H_2_O_2_ accumulation in leaves was detected by DAB staining and H_2_O_2_ content in leaves in wild type and transgenic *Arabidopsis* under different stresses was measured with Plant H_2_O_2_ Kit. Data are means of three replicates ± SE.

Hydrogen peroxide in leaves was detected *in situ* using 3, 3′-Diaminobenzidine (DAB) histochemical staining method (Fig. 4A). The DAB staining results directly ‘visualized’ the H_2_O_2_ content in the plants based on the density of staining. The color of the rosette leaf showed no difference between WT and *SsMT2*-transgenic plants without heavy metal or salt stresses (Fig. 4A). The accumulation of H_2_O_2_ in plants under stress conditions was detected in both transgenic and non-transgenic plants. The color of the WT leaf was darker than that of the leaves of the *SsMT2*-transgenic line under different stress, which indicated that the H_2_O_2_ content in the transgenic line was lower than that of the WT plant after 48 h treatment (Fig. 4B). *SsMT2* increased the H_2_O_2_ scavenging function of the transgenic plants, indicating that the transgenic plants had better tolerance to oxidative stresses.

## Discussion

The expression of the *SsMT2* gene was increased significantly after *S*. *salsa* plants were grown under various stresses and indicated that the *SsMT2* gene may be involved in adaptation to these stresses. Similar expression pattern of *MTs* was induced when CdCl_2_ was applied^19,20,36^, and when salt stresses presented in plants^23,24,37^. The *SsMT2*-transgenic yeast showed higher tolerance to CdCl_2_, NaCl, and NaHCO_3_ stress than the non-transgenic yeast in present study. In plants, different MTs often showed different expression patterns in different plant organs. For example, type 2 MTs were preferentially expressed in the leaves^11,38^, type 1 MTs were found mainly in roots^39,40^. *SsMT2* was expressed in most organs of *Arabidopsis*, including leaves and seeds, and its expression level increased when the *S*. *salsa* plants were exposed to the stresses conditions. Increased expression implies the *SsMT2* gene transcript may affect plant seed germination and development, which were inhibited under the stressful environments^41,42^. MTs had significant impacts on plant growth when the plant suffered various abiotic stresses^40,43^. In this study, transgenic *Arabidopsis* plants had significantly higher seed germination rates and more vigorous seedling growth than non-transgenic plants under high concentrations of metals, salts or hydrogen peroxide. These results indicated that the *SsMT2* gene was involved in the transgenic *Arabidopsis* accommodation of metal, salt and/or oxidant stresses.

*SsMT2* transgenic yeast and *Arabidopsis* plants increased tolerance to CdCl_2_ stress. However, Cd^2+^ accumulation in cells were elevated and indicated that the *SsMT2* expression and Cd^2+^ accumulation have positive linear correlation. The *SsMT2* gene has the same function with the *CeMT2b* gene, which greatly increased Cd^2+^ tolerance and Cd^2+^ accumulation in *E*.*coli* and tobacco^44^. *Arabidopsis* MT1 knock-down lines were hypersensitive to Cd^2+^ and accumulated lower amounts of Cd^2+^ when compared with WT plants^45^. Compared with the wild type, transgenic plants of *Ziziphus jujuba* overexpressing the *ZjMT* gene and accumulate more Cd^2+^ in the roots^43^. However, there are some exceptions, for example, *BcMT2* ^46^ and *TcMT2* ^47^ transgenic lines did not increase tolerance to Cd^2+^ nor did they increase Cd^2+^ accumulation. In this study, the Cd^2+^ accumulation was higher in the transgenic yeast and *Arabidopsis*, and more tolerance to Cd^2+^ than WT plants. The SsMT2 protein chelates the Cd^2+^ in the cytoplasm, and thus blocks Cd^2+^ from freely interacting with cytoplasmic components or entering into organelles. Via this mode of action, decreased Cd^2+^ does limited damage transgenic yeast cells and plants, whereas Cd^2+^ damages WT yeast and plants. The full function of MTs to influence Cd^2+^ tolerance and Cd^2+^ accumulation in cells requires further investigation to elucidate its function.

Sodium ion accumulation in *SsMT2*-overexpressed yeast and plants was significantly lower than that in WTs under high NaCl or NaHCO_3_ environments. There are three mechanisms to prevent excess Na^+^ accumulation in the plant. First, Na^+^ in plant cells may be reduced once Na^+^ influx transporter genes are activated. Second, Na^+^ can be transported and stored in vacuoles. Third, Na^+^ in the cytoplasm can be exported to external medium or the apoplast via plasma membrane Na^+^/H^+^ antiporters^48^. The plant MTs do not contain signal peptides and do not have Na^+^ transportation function. The reason for resulting in lower Na^+^ concentration in *SsMT2*-transgenic lines and enhancing the tolerance of transgenic organism to salt stress may be that the *SsMT2* gene interacted with transporter genes. Overexpression of *SsMT2* in transgenic lines induced the transport Na^+^ out of plant. Lower Na^+^ concentration in the *SsMT2*-transgenic lines probably decreased damage to the plant and increased the tolerance of transgenic yeasts and plants to Na stress.

The exposure of plants to heavy metals and salts can induce ROS to be produced and thus change the balance between ROS production and scavenging^49,50^. *SsMT2*-transgenic lines improved H_2_O_2_ tolerance in both transgenic yeast and *Arabidopsis* plants. Compared with WT plants, *SsMT2*-transgenic *Arabidopsis* plants produced less H_2_O_2_. This observation was consistent with the results of MTs in other plant species, such as *Arabidopsis* T-DNA insertion mutant *mt2a* ^51^, *E*. *haichowensis EhMT1* gene^12^, *Casuarina glauca CgMT1* gene^52^, and Gossypium hirsutum *GhMt3a* ^13^ the transgenic seedlings of these species had less H_2_O_2_ than that in control plants under various stresses. The *SsMT2* gene is involved in the mediation of H_2_O_2_ scavenging during the abiotic stress and resulted in much lower level of H_2_O_2_ accumulated in the transgenic plants. Therefore, the *SsMT2* gene plays an important role in reactive oxygen species scavenging under the stresses imposed in this study. The present study also provided evidence that *SsMT2* may decrease the impact by induced H_2_O_2,_ and protected plants from damage.

In conclusion, *SsMT2* was expressed from seed germination and increased tolerance to stress in transgenic plants. H_2_O_2_ content in transgenic lines was lower than the control. These results suggest that the role of *SsMT2* to influence plant or yeast tolerance to heavy metal and salt stresses may directly bind ion and trigger other genes’ function, or indirectly improve ROS-scavenging ability.

## Materials and Methods

### Cloning of full-length open reading frame (ORF) region of *SsMT2*

We have identified some candidate salt-responsive genes in *S*. *salsa* using the full-length cDNA over-expressing gene (FOX)-hunting system^53^. The *SsMT2* gene was one of those genes identified. Seeds of *S*. *salsa* plants were collected from an alkaline soil area in Northeast China and germinated on MS medium^54^ at 28 °C under 2000 Lux irradiation with a 16 h light/8 h dark photoperiod in an illuminated incubator. Total RNA was isolated from 4-week old seedlings using RNeasy Plant Mini Kit (Qiagen, Hilden, Germany). cDNA was synthesized from l µg of the total RNA with Prime-Script Reverse Transcriptase (Takara, Tokyo, Japan) using an oligo (dT) primer. MT cDNA sequence from FOX-hunting system was obtained and open reading frame (ORF) was found by blasting in the NCBI database. A transcript fragment was amplified by PCR from the cDNA with the forward primer (5′-ATGTCTTGCTGTGGTGGTAACTGTGG-3′) and reverse primer (5′-TCATTTGCAGGTGCATGGGTTG-3′), which were designed from the MT ORF sequence. The PCR product was purified from agarose gel using the DNA Gel Extraction Kit (Generay, Shanghai, China) and cloned into plasmid pMD18-T (Takara, Tokyo, Japan) and sequenced. A new gene was designated as *SsMT2* and its ORF nucleotide sequence and protein sequence was deposited to GenBank database (MF447531).

### Construction of expression and transformation vectors

#### Construction of yeast expression and transformation vectors

The coding region of the *SsMT2* gene was amplified from pMD18T-SsMT2 plasmid DNA with *Bam*HI sense primer 5′-GGATCCATGTCTTGCTGTGGTGGTAA-3′ (restriction site underlined for all restriction enzymes below) and *Xho*I antisense primer 5′-CTCGAGTCATTTGCAGGTGCATGGGT-3′. The PCR amplified fragments were digested with two restriction enzymes *Bam*HI and *Xho*I and then ligated into the *Bam*HI/*Xho*I sites of the vector pYES2 (Takara, Tokyo, Japan) to get pYES2-SsMT2 construct. The plasmid DNA of pYES2-SsMT2 was transformed into competent yeast strain INVSc1 (*S*. *cerevisiae*) (Takara, Tokyo, Japan) using the electric impulse method following the manufacturer’s instructions (Invitrogen)and the transformants were selected based on their growth on uracil deficient synthetic complete (SC-Ura) solid medium (6.7 g/L Yeast Nitrogen Base, 0.77 g/L -Ura Do supplement, PH = 5.8).

#### Construction of plant expression and transformation vectors

The coding region of *SsMT2* gene was amplified from pMD18T-SsMT2 plasmid DNA with the previously described *Bam*HI sense primer and *Sac*I antisense primer 5′-GAGCTCTCATTTGCAGGTGCATGGGT-3′. The PCR fragments were digested with *Bam*HI and *Sac*I and then ligated into the *Bam*HI/*Sac*I site of pBI121 binary vector (Takara, Tokyo, Japan), the plasmid DNAs of pBI121-SsMT2 was transformed into the *Agrobacterium tumefaciens* strain EHA105 (Takara, Tokyo, Japan) and then *Arabidopsis* (ecotype: Columbia) was transformed using the floral dip method^55^.

### Northern blot analysis for the *SsMT2* gene expression in *S*. *salsa*

To examine the expression pattern of the *SsMT2* gene in different organs of *S*. *salsa* plant, total RNA was isolated from roots, leaves, shoots, flowers and seeds respectively using RNeasy Plant Mini Kit (Qiagen, Hilden, Germany) according to the manufacturer’s instructions. Amounts of 5 µg total RNA were fractionated on 1% agarose-formaldehyde gel and transferred onto Hybond N^+^ membranes (Amersham Pharmacia). Hybridizations were carried out at 50 °C using a DIG-labeled probe in hybridization buffer (7% SDS, 50% formamide, 50 mM phosphoric acid buffer (pH = 7.0), 0.9 M NaCl, 0.09 M Sodium citrate), which is the PCR production of the *SsMT2* ORF full length sequence amplified with the forward primer (1 μL,10 μM 5′-ATGTCTTGCTGTGGTGGTAACTGTGG-3′) and reverse primer (1 μL,10 μM 5′-TCATTTGCAGGTGCATGGGTTG-3′), using 10 × PCR digoxigenin (DIG) Labeling Mix (Roche Diagnostics, Switzerland), 0.5 μL Ex-tag, 5 μL Ex-tag buffer, 35.5 μL ddH_2_O. Hybridization signals were detected with CDP-Star (Tropix) using Biotech Image Master VDS-CL Multi-function Bio-imaging Station.

The *SsMT2* gene expression level in *S*. *salsa* seedling under different stresses was detected by Northern blot. The seeds of *S*. *Salsa* were sown onto the MS medium, then the 4-week-old *S*. *Salsa* seedlings were treated with various stresses (100 µM CdCl_2_, 180 µM CdCl_2_, 100 mM NaCl, 150 mM NaCl, 2 mM NaHCO_3_ or 4 mM NaHCO_3_, 1 mM H_2_O_2_ and 5 mM H_2_O_2_) for 48 h.Total RNA was isolated from leaves. Northern blot was conducted as above procedure.

### Stress tolerance of the transgenic yeast

The expression of *SsMT2* gene in transgenic yeast was analyzed using Northern blot. Total RNA from yeast was extracted using the RNeasy Yeast Mini Kit (Qiagen, Hilden, Germany) according to the manufacturer’s instruction. Northern blot was conducted as above procedure.

Western blot was used to investigate the SsMT2 protein amount in yeast. Protein extraction from yeast followed Zhang’s protocol^56^. Yeast cells (1.5 mL in YPG) were harvested prior to stationary phase (OD_600_ = 1.0) by centrifugation. Cells were first pre-treated with 2 M LiAc and then treated with 0.4 M NaOH for 5 min on ice. Finally, cells were centrifuged and yeast whole proteins were extracted with SDS-PAGE sample buffer. Western blot was conducted according to Ohkuni’s protocol^57^. Equal volume of samples was lysed in SDS sample buffer. These samples were separated by 12% SDS-PAGE and subsequently transferred the proteins from gel to a polyvinylidene difluoride (PVDF) membrane using a transfer apparatus at 30 V for 90 min. After blocked in PBST (Phosphate Buffered Saline with Tween20) containing 5% skimmed milk for 1 h at room temperature, membrane was incubated with MT antibody (1: 3,000) overnight at 4 °C and wash membrane 3 times for 10 min each time with 1x PBST, then incubated with alkaline phosphatase-conjugated goat anti-rabbit immunoglobulins (1: 5,000; Sigma) at 37 °C for 1 h. Wash membrane 3 times for 10 min each time with 1x PBST. The signals were detected with CDP-Star detection reagent using Biotech Image Master VDS-CL Multifunction Bio-imaging Station.

Cells of transgenic yeast harboring pYES2-SsMT2 and pYES2 (control) were respectively incubated in YPG medium at 30 °C overnight. The concentration of overnight culture was adjusted to OD_600_ = 0.5. Culture solutions with serial dilutions (10, 10^−1^, 10^−2^, 10^−3^, and 10^−4^) were spotted onto YPG agar plates which were supplemented with different concentrations of metal (140 µM CdCl_2_ or 160 µM CdCl_2_), salts (600 mM NaCl, 1 M NaCl, 22 mM NaHCO_3_, or 26 mM NaHCO_3_), and oxidant (2.8 mM H_2_O_2_ or 3.2 mM H_2_O_2_), respectively. Photos were taken between the 3^rd^ and 7^th^ day after the stress treatments.

### Stress tolerance of the transgenic *Arabidopsis*

The southern hybridization of genomic DNA of transgenic *Arabidopsis* was conducted to investigate the copy number of the *SsMT2* gene in the transgenic lines. Genomic DNA from 2-week-old *Arabidopsis* (wild type, transgenic lines#1, #2, #3, #4, #5 and #6) leaves was isolated using the CTAB method and then digested with *Hind*III at 37 °C for 60 min. The digested fragments were separated on 1% (w/v) agarose gel and then transferred to the hybrid. Expression of *SsMT2* gene in transgenic *Arabidopsis* was analyzed using Northern blot. The *SsMT2* gene expression level in transgenic *Arabidopsis* lines (#1, #5 and #6) was detected by Northern blot.

The seeds of wild type and the third generation(homozygous) transgenic *Arabidopsis* plants (#1, *#*5, #6) were surfaced-sterilized with 70% ethanol for 1 min, followed by 1% NaClO solution for 3 min, and then rinsed three times in sterile water. The seeds were sown onto agar plates that contained MS basal medium, 1% (w/v) sucrose, and 0.8% (w/v) agar, supplemented with either filter-sterilized 100 µM CdCl_2_, 180 µM CdCl_2_, 100 mM NaCl, 150 mM NaCl, 2 mM NaHCO_3_, 4 mM NaHCO_3_, 1 mM H_2_O_2_ or 5 mM H_2_O_2_. Seeds germinated on MS medium were used as control, three times repeat. Photos were taken on the 14^th^ day after the stress treatments. Germination rate was calculated when the transgenic and wild type *Arabidopsis* no longer sprouted.

To find out whether the *SsMT2* gene impacts the early seedling development under the different stresses, the seeds of wild type and transgenic *Arabidopsis* (#1, #5) were germinated on MS medium. The 14-day-old seedlings were transplanted onto MS medium (as a control) and MS medium supplemented with different concentrations of metals (100 µM CdCl_2_, 180 µM CdCl_2_), salts (100 mM NaCl, 150 mM NaCl, 2 mM NaHCO_3_, 4 mM NaHCO_3_), or oxidant (1 mM H_2_O_2_, 5 mM H_2_O_2_), respectively. The plates were positioned vertically on shelves in order to compare root growth visually. Root length and dry weight were measured after stresses applied 7^th^ and 14^th^ day, three times repeat. Photos were taken between the 7^th^ and 14^th^ day after the stress treatments.

In addition, we examined the stress tolerance at the plant adult stage. Briefly, wild type and transgenic seeds (#1, #5) were grown on MS medium. One-week-old plants were transferred to pots filled with 3:1 mixture of nutrition soil: peat in a chamber (22 °C, 100 M photons·m^−2^·s^−1^, 60% relative humidity, 16/8 h day-night cycles). The soil-grown plants were watered with 50 mM CdCl_2_, 100 mM CdCl_2_, 400 mM NaCl, 500 mM NaCl, 400 mM NaHCO_3_, 500 mM NaHCO_3,_1.5 M H_2_O_2_, or 2 M H_2_O_2_ solution respectively every 4 days for a total of 12 days, three times repeat. The plants survived rate was calculated on the 12^th^ day after treatment and we took photos at the same time.

### Ion uptake in transgenic yeast

To examine whether the *SsMT2* gene involves in the accumulation of metals in yeast cells, the Cd^2+^ or Na^+^ content was measured with the method previously described^58^. In brief, yeast cells cultured in the YPG liquid medium containing 140 µM CdCl_2_, 160 µM CdCl_2_, 600 mM NaCl, 1 M NaCl, 22 mM NaHCO_3_ or 26 mM NaHCO_3_ and maintained at 30 °C with shaking at 160 rpm for 12 h. After treatment, 200 mg (dry weight) of cells were collected and analyzed using atomic absorption spectrophotometer (AA800, Perkin Elmer, America). Blank sample was used 10 times to calculate the standard deviation, then the measured standard deviation value was put into the regression equation to figure out that Atomic Absorption Spectrometry (AAS) detection limitation for Cd^2+^ was 0.0003 μg/g and Na^+^ was 0.0005 μg/g. The samples were divided into two groups, two samples per group. In each group, one sample was added the standards, another one as a control. Every time the two samples were measured in parallel. The recovery rate was calculated according to the additive amount and the detectable quantity of the ions. The recovery rate for Cd^2+^ in the standard reference material (GSB 04-1721-2004 Beijing, China) was 95% and Na^+^ in the standard reference material (GSB 04-1738-2004 Beijing, China) was 98%, indicating that this method is accurate.

### Ion uptake in *Arabidopsis* plants

Fourteen-day-old WT and transgenic *Arabidopsis* plants (#1 transgenic plant) were treated without (control) or with each of following solution: 100 µM CdCl_2_, 180 µM CdCl_2_, 100 mM NaCl, 150 mM NaCl, 2 mM NaHCO_3_ or 4 mM NaHCO_3_ respectively for 48 h. Roots and shoots were harvested and washed in deionized water. Desorption of shoot and root was performed with 1 mM MES-Tris (pH 6.0) containing 0.5 mM CaCl_2_. The samples were dried at 80 °C for 2 days for dry weight measurement. The dried plant materials were digested in a 5 mL mixture of HNO_3_ and HClO (87:13, v/v) overnight at room temperature, diluted with 5 mL of 2.5% HNO_3_, and then measured for ion contents by an atomic absorption spectrophotometer.

### Reaction to H_2_O_2_ stress in transgenic *Arabidopsis* plants

Fourteen-day-old WT and transgenic *Arabidopsis* plants (#1) were treated without (control) or with each of 100 µM CdCl_2_, 180 µM CdCl_2_, 100 mM NaCl, 150 mM NaCl, 2 mM NaHCO_3_, 4 mM NaHCO_3_, 1 mM H_2_O_2_ or 5 mM H_2_O_2_ respectively for 48 h. H_2_O_2_ accumulation in plant leaves was visualized by histochemical staining with 3, 3′-Diaminobenzidine (DAB). DAB is oxidized by H_2_O_2_ in presence of peroxidases and produces reddish brown precipitate^59^. The treated leaves were immersed in 1 mg·mL^−1^ DAB solution, vacuum-infiltrated for 10 min, and then incubated at room temperature for 12 h in the absence of light until the appearance of blown spots. The stain solution was poured off and the chlorophyll was removed by incubating the samples in absolute ethanol overnight. Staining of the rosette leaf was photographed with a microscopy (Olympus). The H_2_O_2_ content was also measured using Plant H_2_O_2_ ELISA Kit (America Rapid Bio).

### Statistical analysis

All treatments were arranged in a randomized complete block design with three replicates and subjected to analysis of variance. The differences among the mean values of different treatments were compared using Duncan’s Multiple Range tests at significant difference level of *P* ≤ 0.05 using SPSS (Statistical Product and Service Solutions) for Windows version 11.5.

## Electronic supplementary material

Supplementary Information
