# Supplementary material for: Functional characterization of a type 2 metallothionein gene, SsMT2, from alkaline-tolerant Suaeda salsa
Source: Sci Rep. 2017 Dec 20;7:17914. doi: 10.1038/s41598-017-18263-4 (PMC5738349; doi:10.1038/s41598-017-18263-4)
Supplement: Supplementary file 1 — Supplementary Information [file 41598_2017_18263_MOESM1_ESM.doc]

**Functional characterization of a type 2 metallothionein gene, *SsMT2*, from alkaline-tolerant *Suaeda salsa***

Shumei Jin^1#^, Chang Xu^1#^, Guoliang Li^3^, Dan Sun^1,4^, Ying Li^1^, Xinwang Wang^2^*, Shenkui Liu^1^*

**Affiliations:**

^1^Key Laboratory of Saline-alkali Vegetation Ecology Restoration in Oil Field (SAVER), Ministry of Education, Alkali Soil Natural Environmental Science Center (ASNESC), Northeast Forestry University, Harbin, China 150040

^2^ USDA-ARS, Southern Plains Agricultural Research Center, College Station, TX, 77845.

^3^ Institute of Maize, Heilongjiang Academy of Agricultural Sciences, Harbin, China150086

^4^ Institute of Vegetables and Flowers, Chinese Academy of Agricultural Sciences, Beijing, China100081

# First two authors equally contributed to this project

***Correspondence:**

Shenkui Liu, Ph.D. E-mail: shenkuiliu@nefu.edu.cn，

or

Xinwang Wang, Ph.D. E-mail: xinwang.wang@ars.usda.gov

Key Laboratory of Saline-alkali Vegetation Ecology Restoration in Oil Field (SAVER), Ministry of Education, Alkali Soil Natural Environmental Science Center (ASNESC), Northeast Forestry University, Harbin, China 150040, TEL: 86-0451-82191402

**Supplementary Information**

**Figure legends.**

**Fig.S1** Multiple sequence alignment of the amino acid sequences of *Suaeda salsa* *SsMT2* gene with genes *SbMT* (JF780913) in *Salicornia brachiata*, *AcMT* (AF268027) in *Amaranthus cruentus*, *SnMT* (ADP92404) in *Silene niceensis*, and *SmMT* (ABR92329) in *Salvia miltiorrhiza*.


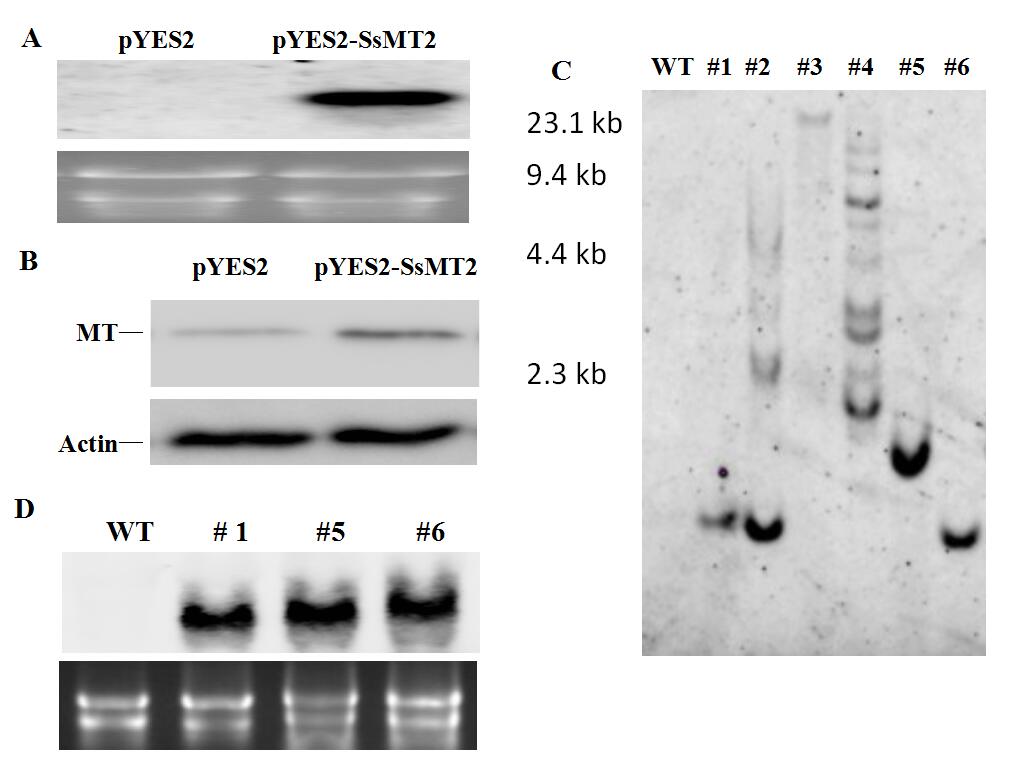


**Fig.S2** *SsMT2* gene expression and copies were presented by Northern blot, Western blot and Southern blot respectively in transgenic yeast and *Arabidopsis*.

(**A**). *SsMT2* gene expression in transgenic yeast using Northern blot analysis. One distinct band was detected in the transgenic yeast and no signal in the control, which indicated that the *SsMT2* gene was expressed in the transgenic yeast. pYES2 is a control without *SsMT2* gene and pYES2-SsMT2 is the transgenic yeast. Cropped images were displayed and original blots are shown in the figure Supplementary 4.

(**B**). The MT protein quantification in yeast using Western blot analysis. The weaker signals were detected without *SsMT2* yeast and the stronger signals were observed at *SsMT2* transformed yeast by western blot. This result indicated that some other MT protein present in the yeast, and *SsMT2* transformed yeast has more MT protein than untransformed yeast. pYES2 is a control without *SsMT2* gene and pYES2-SsMT2 is the transgenic yeast. Cropped images were displayed and original blots are shown in the figure Supplementary 4. (**C**). *SsMT2* gene copies in six third-generation transgenic *Arabidopsis* plants using Southern blot analysis. Southern blot showed one and more distinct bands in the transgenic *Arabidopsis*, with one copy in four lines (#1, #3, #5 and #6) and three copies (#2) and nine copies (#4). No positive signal was detected in the wild type (WT) *Arabidopsis*. WT, wild-type plant is negative control. #1, #2, #3, #4, #5 and #6 are transgenic plants. (**D**). *SsMT2* gene expression in transgenic *Arabidopsis* plants using Northern blot analysis. One distinct band was detected in the transgenic plants and no signal in the wild-type plants. WT, wild-type plant is negative control. #1, #5 and #6 are transgenic plants. Cropped images were displayed and original blots are shown in the figure Supplementary 4.


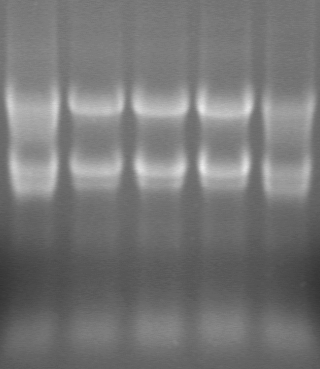

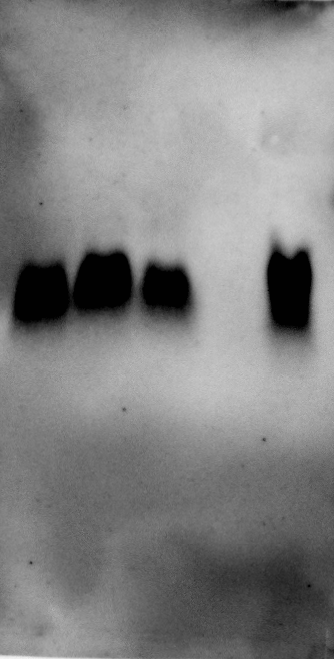

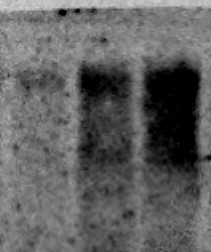

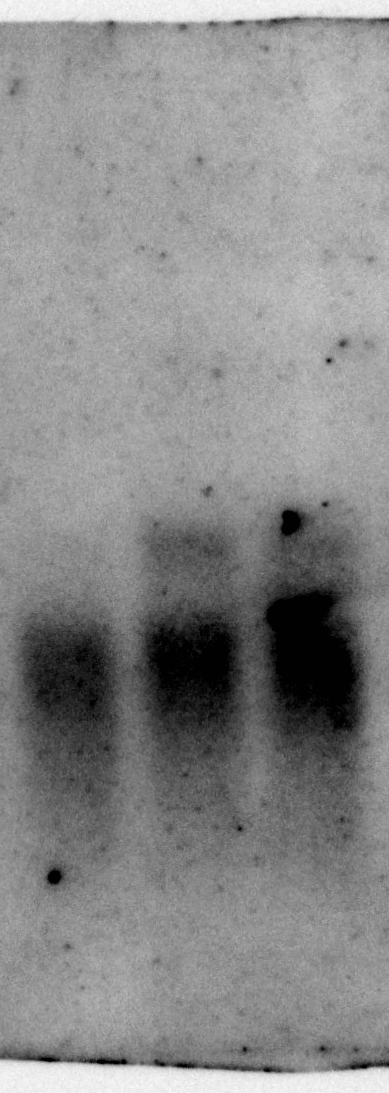

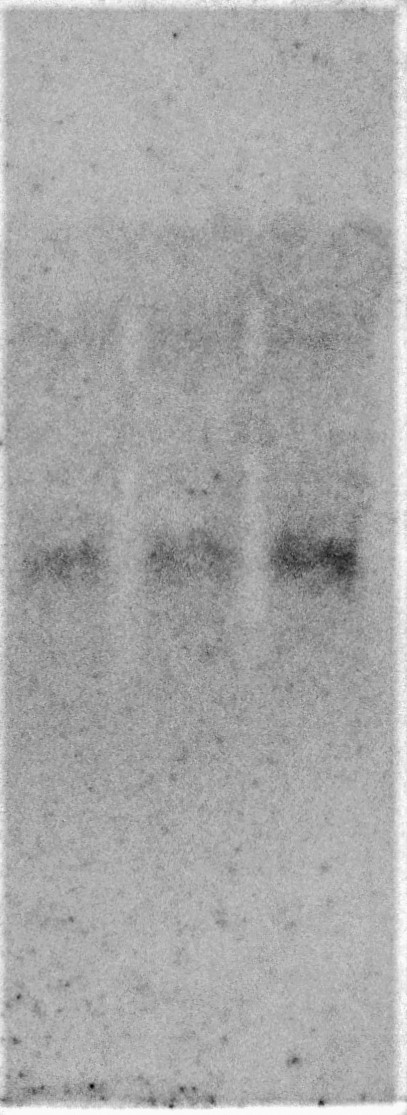

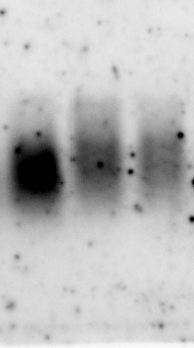

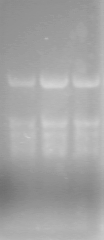

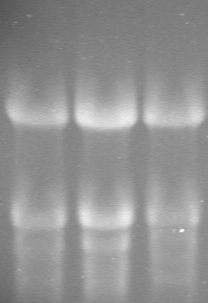

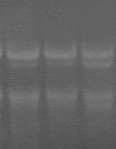

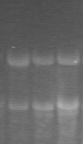


Fig 1B

Fig 1A

**Fig.S3.** Original blot for Fig 1


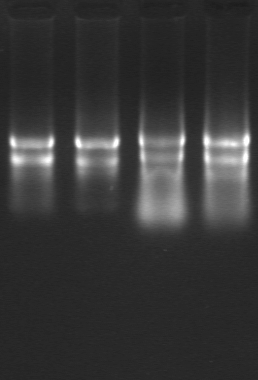

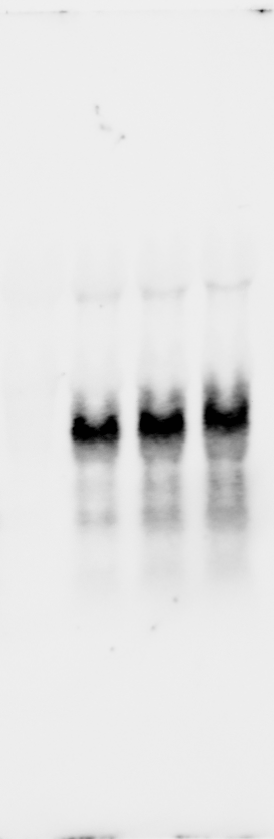

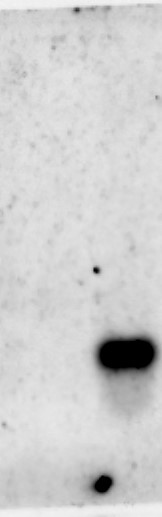

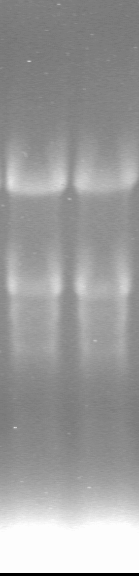

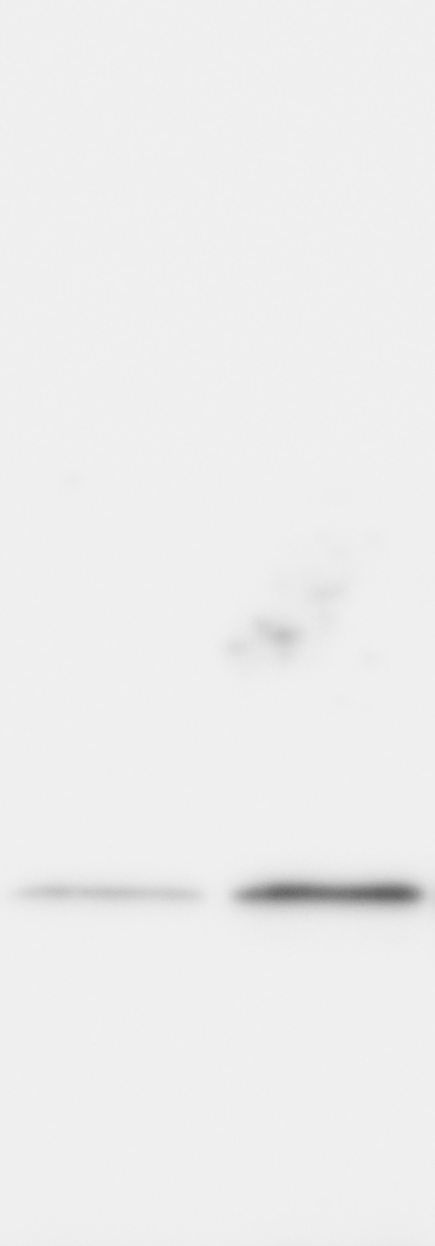


Fig S2A

Fig S2D

Fig S2B


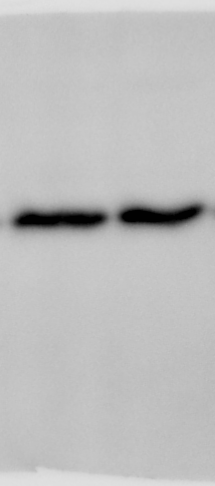


**MT—**

**Actin—**

**Fig.S4.** Original blot for Fig. S2A、B and D
